# Closed system RT-qPCR as a potential companion diagnostic test for immunotherapy outcome in metastatic melanoma

**DOI:** 10.1186/s40425-019-0731-9

**Published:** 2019-09-18

**Authors:** Swati Gupta, Leena McCann, Yvonne G. Y. Chan, Edwin W. Lai, Wei Wei, Pok Fai Wong, James W. Smithy, Jodi Weidler, Brian Rhees, Michael Bates, Harriet M. Kluger, David L. Rimm

**Affiliations:** 1 Department of Pathology, Yale University School of Medicine, 310 Cedar Street, PO Box 208023, New Haven, CT 06510 USA; 2 Oncology Research and Development, Cepheid, Sunnyvale, CA USA; 3 Department of Biostatistics, Yale School of Public Health, New Haven, CT USA; 4 Department of Medicine, Brigham and Women’s Hospital, Boston, MA USA; 5 Medical and Scientific Affairs and Strategy, Oncology, Cepheid, Sunnyvale, CA USA; 6 Department of Internal Medicine (Medical Oncology), Yale University School of Medicine, New Haven, CT USA

## Abstract

**Background:**

In melanoma, there is no companion diagnostic test to predict response to programmed cell death 1 (PD-1) axis immune checkpoint inhibitor (ICI) therapy. In the adjuvant setting, only one in five patients may benefit from ICI, so a biomarker is needed to select those that may or may not benefit. Here, we test a new 4-gene multiplex immunotherapy panel with research use only (RUO) prototype mRNA expression profile on the GeneXpert closed system using real-time quantitative reverse transcription polymerase chain reaction (RT-qPCR) for association with clinical benefit after treatment with ICI therapy in metastatic melanoma patients.

**Methods:**

Pretreatment formalin-fixed paraffin-embedded (FFPE) tissue sections from melanoma patients treated with anti-PD-1 therapy (pembrolizumab, nivolumab, or ipilimumab plus nivolumab) between 2011 and 17 were selected from the Yale Pathology archives. FFPE sections were macrodissected to enrich for tumor for quantitative assessment of *CD274 (PD-L1), PDCD1LG2 (PD-L2), CD8A,* and *IRF1* by RT-qPCR multiplex mRNA panel. Multiplex panel transcript levels were correlated with clinical benefit (complete response [CR], partial response [PR], stable disease [SD]); disease outcomes (progression-free survival [PFS] and overall survival [OS]); and protein levels assessed by quantitative immunofluorescence (QIF).

**Results:**

Transcript levels were significantly higher in responders (CR/PR/SD) than in nonresponders (PD) for *CD8A* (*p* = 0.0001) and *IRF1* (*p* = 0.0019). PFS was strongly associated with high *CD274* (*p* = 0.0046), *PDCD1LG2* (*p* = 0.0039), *CD8A* (*p* = 0.0002), and *IRF1* (*p* = 0.0030) mRNA expression. Similar associations were observed for OS with high *CD274* (*p* = 0.0004), *CD8A* (*p* = 0.0030), and *IRF1* (*p* = 0.0096) mRNA expression. Multivariate analyses revealed significant PFS and OS associations with immunotherapy panel markers independent of baseline variables. Exploratory analyses revealed a novel significant association of high combined *CD274* & *PDCD1LG2 (L1/L2*) transcript expression with PFS (*p* < 0.0001) and OS (*p* = 0.0011), which remained significant at a multivariate level for both PFS (HR = 0.31) and OS (HR = 0.39).

**Conclusions:**

Individual immunotherapy panel markers *CD274, PDCD1LG2, CD8A*, *IRF1* and a combined *L1/L2* mRNA levels show promising associations with melanoma immunotherapy outcome. The turnaround time of the test (2 h) and easy standardization of the platform makes this an attractive approach for further study in the search for predictive biomarkers for ICI.

## Background

Immune checkpoint blockade (ICI) antibodies targeting cytotoxic T-lymphocyte antigen 4 (CTLA-4) and programmed cell-death protein 1 (PD-1) have shown compelling efficacy in more than 15 cancer types [1]. In advanced melanoma durable response rates (i.e., > 2 years) for three U.S. Food and Drug Administration (FDA) approved immune checkpoint inhibitor antibodies, ipilimumab (anti-CTLA-4), anti-PD-1 (pembrolizumab and nivolumab), and combination of ipilimumab and nivolumab are 11–15, 33–45 and 60% respectively [2, 3]. However, majority of the patients do not respond to monotherapy regime and a subset of patients develop severe adverse events with combination regime [4–7].

In advanced melanoma, PD-L1 IHC 28–8 pharmDx assay is FDA approved as a complementary diagnostic for nivolumab [2, 8]. PD-L1 positive patients are more likely to respond to anti-PD-1 axis ICI than PD-L1 negative patients [9, 10]. However, the predictive value of PD-L1 expression by IHC in melanoma is controversial, as PD-L1 positive melanoma patients also show better survival in chemotherapy arm [11]. Furthermore, PD-L1 expression in melanoma is low, difficult to measure and quite heterogeneous [12]. Moreover, PD-L1 detection by IHC has major limitations, such as lack of standardization with different antibodies, various cutoffs for scoring and defining positivity [9, 13, 14]. Thus, in metastatic melanoma, there is no companion diagnostic test that can predict response to anti-PD-1 axis immune checkpoint inhibitor therapy.

In the adjuvant setting, only 1 in 5 patients benefit from ICI. There are also relatively severe and prevalent adverse events for a population that may be surgically cured. Thus, there is a more compelling need for a companion diagnostic test in the adjuvant setting than in the metastatic setting. Here, we test a new 4-gene multiplex immunotherapy panel (*CD274, PDCD1LG2, CD8A,* and *IRF1*) with research use only (RUO) prototype mRNA expression profile on the GeneXpert closed system using real-time quantitative reverse transcription polymerase chain reaction (RT-qPCR) for association with clinical benefit after treatment with ICI in metastatic melanoma patients toward the goal of a sensitive and specific test for prediction of benefit from ICI.

## Methods

### Patient cohort

Patient cohort is a retrospective collection of 116 melanoma patients treated with anti-PD-1 therapy from 2011 to 17 at Yale. Pretreatment formalin-fixed, paraffin-embedded (FFPE) specimens were reviewed by a board-certified pathologist. The specimens included 78 resections and 38 biopsies. Data were collected from clinical records and the cut-off date was September 2017. A summary of cohort characteristics is detailed in Table 1. All patients provided written informed consent or waiver of consent. The study was approved by the Yale Human Investigation Committee protocol #9505008219.

**Table 1:** Clinicopathological characteristics of the melanoma cohort treated with anti-PD-1 therapy

Characteristic	Anti-PD-1 patients, No. (%)	Objective response rate (CR/PR), No. (%)	Disease control rate (CR/PR/SD), No. (%)
Overall	116 (100)	54 (47)	80 (69)
Age (y)			
< 65	66 (57)	33 (61)	50 (62)
≥ 65	50 (43)	21 (39)	30 (38)
Sex			
Male	69 (59)	34 (63)	47 (58)
Female	47 (41)	20 (37)	33 (42)
Treatment			
Pembrolizumab	41 (35)	20 (37)	30 (38)
Nivolumab	18 (16)	7 (13)	9 (11)
Ipilimumab plus nivolumab	57 (49)	27 (50)	41 (51)
Prior immune checkpoint blockade			
Yes	36 (31)	14 (26)	23 (29)
No	80 (69)	40 (74)	57 (71)
Mutation status			
BRAF	39 (33)	19 (35)	27 (34)
NRAS	18 (16)	8 (15)	11 (14)
KIT	2 (2)	1 (2)	2 (2)
None detected	57 (49)	26 (48)	40 (50)
Stage at diagnosis			
I	24 (21)	14 (26)	19 (24)
II	23 (20)	12 (22)	16 (20)
III	38 (32)	16 (30)	24 (30)
IV	20 (17)	6 (11)	13 (16)
Not available	11 (10)	6 (11)	8 (10)

### Quantitative multiplex RT-PCR

Quantitative multiplex RT-qPCR was performed using GeneXpert (GX) system. Briefly, 5 μM thick FFPE tissue sections were collected and macrodissected to collect tumor. Samples were mixed with 5 μl Proteinase K and 260 μl FFPE lysis reagent. After a 30-min incubation at 80 °C, 260 μL of > 95% ethanol was added to the lysed samples and vortexed to mix. This mixture was transferred to the cartridge and was run on the GX system. This assay isolates the total RNA, performs a 1-step RT-PCR and provides Ct values for the endogenous control, *POLR2J*, and the target genes, *CD274*, *PDCD1LG2*, *CD8A* and *IRF1*. Results were expressed as a delta cycle threshold (dCt) value, defined as the Ct of the control gene, *POLR2J*, minus the Ct of each of the target genes (*CD274*, *PDCD1LG2*, *CD8A* and *IRF1*). Median values for each marker were used to define high versus low mRNA expression group. For combined *CD274* & *PDCD1LG2* (*L1/L2*) transcript data, we added 10 to individual dCt values of both the transcripts followed by their addition “[*CD274* (dCt) + 10] + [*PDCD1LG2* (dCt) + 10]”. X-Tile software was used to determine thresholds to define low and high statuses for the *L1/L2* transcript data [15].

### Statistical analysis

Inter-transcript regression was assessed using nonlinear exponential growth equation (*R*^*2*^). Response Evaluation Criteria in Solid Tumors (RECIST) 1.1 were used to determine best overall response as complete response (CR), partial response (PR), stable disease (SD), or progressive disease (PD). Disease control rate (DCR; CR/PR/SD) were correlated with multiplex RT-qPCR immunotherapy panel transcript expression using two-tailed unpaired Student’s *t*-tests. Receiver operating characteristic (ROC) curves measured the predictive performance of transcript expression. Kaplan–Meier estimates of progression-free survival (PFS) and overall survival (OS) functions were compared using the log-rank test. Multivariable Cox proportional hazards model included age, sex, mutation status, stage, treatment, and prior ICI as covariates and analyses were carried out using JMP Pro v13.0 (SAS Institute Inc., Cary, NC) statistical analysis software. All data sets were analyzed and plotted using GraphPad Prism v7.0 software for Windows (GraphPad Software, Inc., La Jolla, CA). *P* values less than 0.05 were considered statistically significant.

## Results

### Inter-transcript regression of immunotherapy markers for melanoma

**Fig. 1:**
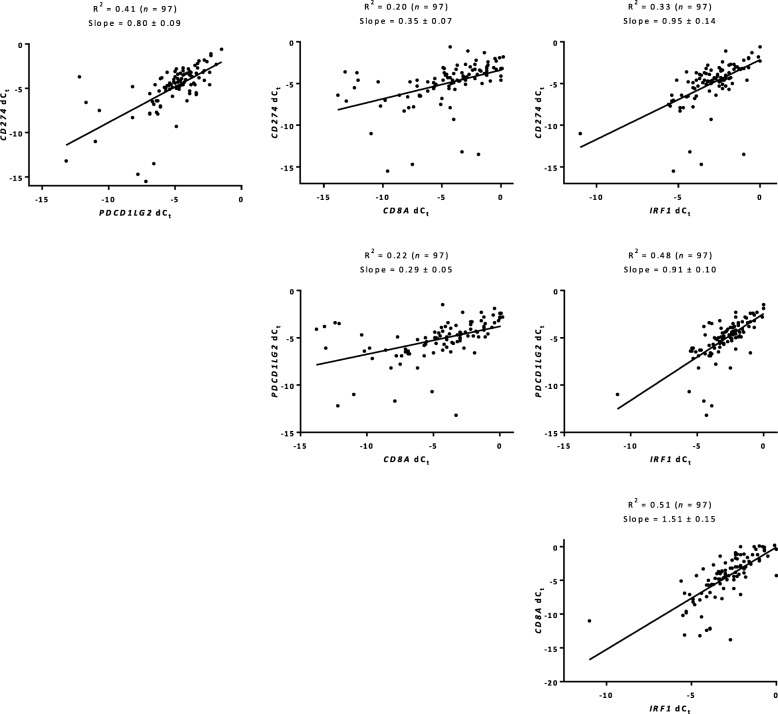
Inter-transcript regressions in melanoma. Relationship between four transcripts, *CD274*, *PDCD1LG2*, *CD8A*, and *IRF1* as determined by multiplex RT-qPCR immunotherapy panel in melanoma patients treated with anti-PD-1 therapy

To assess the mRNA expression of four immunotherapy markers, *CD274*, *PDCD1LG2*, *CD8A* and *IRF1*, we used a multiplex RT-qPCR immunotherapy panel on the GeneXpert platform in melanoma patients treated with anti-PD-1 therapy. Inter-transcript regression for all four immunotherapy markers showed concordance with R^2^ ranging from 0.20 to 0.51 (Fig. 1). Specifically, between *CD274* and *PDCD1LG2* (R^2^ = 0.41); *PDCD1LG2* and *IRF1* (R^2^ = 0.48)*;* and *CD8A* and *IRF1* (R^2^ = 0.51) there was a strong agreement. Regression of transcript (dC_t_) and protein (QIF scores) measurements using nonlinear exponential growth equation showed high concordance with both CD8 (R^2^ = 0.66) and IRF1 (R^2^ = 0.40), but not PD-L1 (R^2^ = 0.05) (Additional file 1: Figure S1).

### Immunotherapy markers predicts response to anti-PD-1 checkpoint blockade in melanoma

**Fig. 2:**
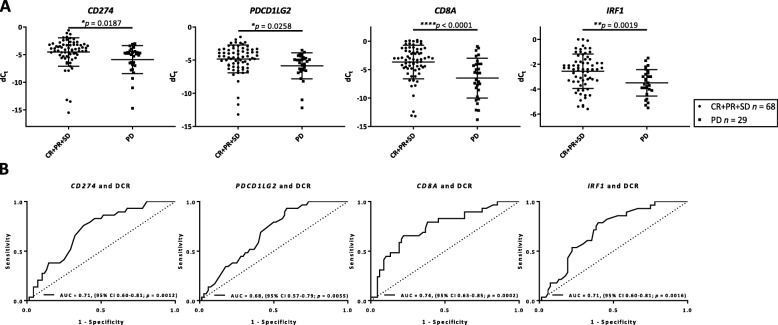
Multiplex RT-qPCR immunotherapy panel markers predicts response to anti-PD-1 checkpoint blockade in melanoma. **a** *CD274*, *PDCD1LG2*, *CD8A*, and *IRF1* transcript expression per RECIST category of DCR. Data are presented as mean with standard deviation (error bars). **b** Predictive performance of *CD274*, *PDCD1LG2*, *CD8A*, and *IRF1* transcript expression by ROC curves in terms of DCR category

Anti-PD-1 responders (CR/PR/SD, *n* = 68) and non-responders (PD, *n* = 29) were identified using RECIST category of DCR. Interestingly, high mRNA expression for each of the four immunotherapy markers, *CD274* (*p* = 0.0187), *PDCD1LG2* (*p* = 0.0258), *CD8A* (*p* < 0.0001) and *IRF1* (*p* = 0.0019) was found to be associated with response to immunotherapy (Fig. 2a). ROC for predictive performance over the range of the transcript expression showed the high discriminatory ability of all four immunotherapy markers. Areas under the ROC curves and their 95% confidence intervals (CIs) were 0.71 (0.60–0.81) for *CD274*, 0.68 (0.57–0.79) for *PDCD1LG2*, 0.74 (0.63–0.85) for *CD8A*, and 0.71 (0.60–0.81) for *IRF1* (Fig. 2b). Similar association using RECIST category of objective response rate were observed for *CD8A* (*p* = 0.0025) and *IRF1* (*p* = 0.0142) with response to immunotherapy with and AUC of 0.70 (0.59–0.80) and 0.65 (0.54–0.76), respectively (Additional File 2: Figure S2).

### Survival outcomes and immunotherapy markers in melanoma

**Fig. 3:**
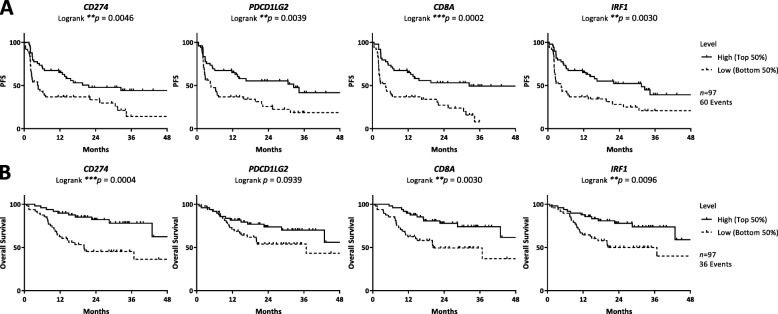
Multiplex RT-qPCR immunotherapy panel and survival outcome of anti-PD-1 treated melanoma patients. Kaplan–Meier analysis of **a** progression-free survival and **b** overall survival of anti-PD-1 treated melanoma patients according to *CD274*, *PDCD1LG2*, *CD8A*, and *IRF1* transcript expression by multiplex RT-qPCR immunotherapy panel. Low and high statuses were defined using median cut point

PFS was strongly associated with high *CD274* (*p* = 0.0046), *PDCD1LG2* (*p* = 0.0039), *CD8A* (*p* = 0.0002), and *IRF1* (*p* = 0.0030) transcript expression (Fig. 3a). Similar associations were observed for OS with high *CD274* (*p* = 0.0004), *CD8A* (*p* = 0.0030), and *IRF1* (*p* = 0.0096) transcript expression (Fig. 3b). Multivariate analyses revealed PFS and OS association with both *CD8A* (PFS: HR 0.39, 95%CI 0.22–0.68, *p* = 0.0009 l; OS: HR 0.40, 95% CI 0.18–0.84, *p* = 0.0152) and *IRF1* (PFS: HR 0.48, 95% CI 0.26–0.86, *p* = 0.0135; OS: HR 0.36, 95% CI 0.16–0.79, *p* = 0.0109) independent of age, sex, stage, mutation, treatment, and prior ICI. In addition, significant association of *CD274* (HR 0.30, 95% CI 0.13–0.66, *p* = 0.0024) only with OS and *PDCD1LG2* (HR 0.49, 95% CI 0.27–0.89, *p* = 0.0179) only with PFS was observed in multivariate analyses (Table 2A).

**Table 2:** Univariate and multivariate Cox regression analyses for progression-free survival and overall survival of melanoma patients and multiplex RT-qPCR immunotherapy panel markers

Variable (HI/LO)	PFS				OS			
	Univariate analysis		Multivariate^a^ analysis		Univariate analysis		Multivariate* analysis	
	HR (95% CI)	*P* value	HR (95% CI)	*P* value	HR (95% CI)	*P* value	HR (95% CI)	*P* value
A								
*CD274*	**0.48** **(0.29–0.81)**	**0.0053**	0.57 (0.32–1.03)	0.0632	**0.29** **(0.13–0.58)**	**0.0004**	**0.30** **(0.13–0.66)**	**0.0024**
*PDCD1LG2*	**0.47** **(0.27–0.79)**	**0.0044**	**0.49** **(0.27–0.89)**	**0.0179**	0.56 (0.28–1.09)	0.0936	0.52 (0.24–1.11)	0.0922
*CD8A*	**0.36** **(0.21–0.61)**	**0.0001**	**0.39** **(0.22–0.68)**	**0.0009**	**0.38** **(0.18–0.75)**	**0.0051**	**0.40** **(0.18–0.84)**	**0.0152**
*IRF1*	**0.47** **(0.28–0.78)**	**0.0035**	**0.48** **(0.26–0.86)**	**0.0135**	**0.41** **(0.20–0.80)**	**0.0094**	**0.36** **(0.16–0.79)**	**0.0109**
B								
*CD274* & *PDCD1LG2*	**0.30** **(0.16–0.57)**	**< 0.0001**	**0.31** **(0.14–0.59)**	**0.0003**	**0.38** **(0.19–0.73)**	**0.0043**	**0.41** **(0.19–0.86)**	**0.0192**

^a^Cox proportional hazards model included age, sex, mutation status, stage, treatment, and prior immune checkpoint blockade as covariates

*P* values highlighted in bold are statistically significant

**Fig. 4:**
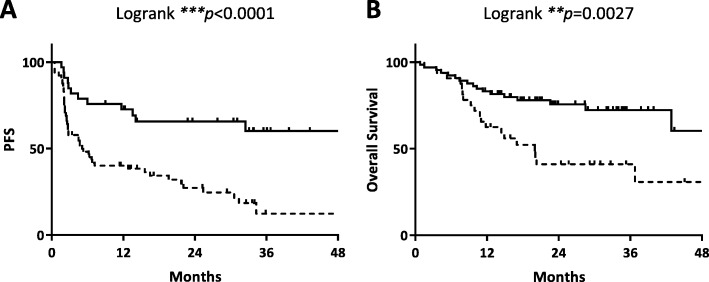
PD-L1 and PD-L2 combination predicts good survival outcome in anti-PD-1 treated melanoma patients. Kaplan–Meier plots of **a** progression-free survival and **b** overall survival of anti-PD-1 treated melanoma patients based on combined *L1/L2* transcript expression by RT-qPCR. Low and high statuses were defined using X-Tile cut point

Since PD-1 antibodies inhibit both the binding of PD-L1 and -L2, and since these were the two mRNAs least correlated in expression, we constructed a signature combining both of these variables. The *L1/L2* combined signature is significantly associated with both PFS (*p* < 0.0001) and OS (*p* = 0.0027) (Fig. 4a-b). Unlike individual *CD274* and *PDCD1LG2* expression, the combination of the expression level of the two mRNAs remained significant at a multivariate level for both PFS (HR 0.31, 95%CI 0.14–0.59, *p* = 0.0003) and OS (HR 0.41, 95%CI 0.19–0.86, *p* = 0.0192) (Table 2B).

## Discussion

The goal of this study was to test a new mRNA approach for association with response and outcome in ICI treated metastatic melanoma. We tested a new 4-gene multiplex immunotherapy panel (*CD274, PDCD1LG2, CD8A,* and *IRF1*) as an mRNA expression profile on the GeneXpert closed system using RT-qPCR. All 4 immunotherapy markers were significantly higher in responders (CR/PR/SD) than in non-responders (PD) and a combined *CD274* & *PDCD1LG2* model showed associations with survival that was independent of age, sex, mutation status, stage, treatment, and prior ICI.

PD-L1 expression by IHC is the most commonly used predictive marker for response to ICI but it has an AUC of around 0.65 in solid tumors [16–19]. Although IHC is currently the only FDA approved method, the marginal predictive power of PD-L1 detection by IHC has been further limited by lack of standardization between different assays and antibodies, various scoring systems and subjectivity in analysis [9, 13, 14]. These weaknesses have been compounded by the success of the assay in different organ systems with different assays which would suggest that a single lab would need to offer multiple non-standardized tests for the same analyte (PD-L1). Detection of mRNA or mRNA signatures on a standardized, internally controlled, close system platform has the potential to address these weaknesses of IHC.

Efforts to predict outcome with mRNA measurements or mRNA signatures have shown some promise. The first and most significant is probably that by Ayers and colleagues that showed that an 18-gene signature performed on the Nanostring platform could predict response to pembrolizumab with an AUC around 0.75 [20]. Chen and colleagues also reported that gene expression profiling using a Nanostring panel is predictive of response in patients that received sequential anti-CTLA4 and anti-PD-1 therapies [21]. Similarly, meta-analysis by Lu and colleagues showed that gene expression profiling had predictive value for solid tumors in response to anti-PD-1, with an AUC of 0.65 [19]. Of note, a recent study by Pare and colleagues demonstrated that PD-L1 transcript alone, measured via Nanostring platform, had moderate correlation with response to single agent anti-PD-1 therapy across multiple tumor types [22]. Another study by Fehrenbacher and colleagues reported the predictive value of 12-gene signature (T-effector and interferon-γ signature) for prolonged OS with Atezolizumab, measured using the Nimblegen platform [23]. In addition, work led by Kowanetz and colleagues showed a 3-gene signature had predictive value for response to Atezolizumab [24]; the signature included *CD274* (PD-L1 mRNA), similar to our efforts. Moreover, we observed that PD-L1 expression by closed system immunotherapy assay could predict the response to immunotherapy with an AUC of 0.71 which is marginally better than IHC.. However, this assay solves a series of major issues associated with PD-L1 IHC including assay variance between vendors, subjective assessment by pathologists, and operator-dependent variation in results. This closed system is objective and operator independent. In summary, while mRNA appears promising, it is too early to determine if this method will gain acceptance in the metastatic setting.

Limited studies have explored the potential role of PD-L2 in predicting response to ICI [10, 25]. Similar to PD-L1, but worse, PD-L2 assessment by IHC has been hampered by lack of validated antibodies and similar IHC issues that have limited PD-L1. Perhaps the most significant effort is that of Yearley and colleagues that showed that high PD-L2 expression was associated with prolonged survival outcome in patients treated with pembrolizumab in Head and Neck Squamous Cell Carcinoma [26]. However, no follow-up data has been published or presented suggesting future use of PD-L2 as a companion diagnostic test.

The secretion of interferon gamma (IFNγ) by infiltrating immune cells including, T, NK, and NK T cells locally activates JAK/STAT signaling in macrophages and dendritic cells [27]. These cells in turn produce chemokines that recruit additional CD8+ T cells. IFNγ also induces synthesis of PD-L1 transcription factor IRF1 and expression of checkpoint inhibitors including PD-L1 and PD-L2 on the surface of tumor, macrophages and dendritic cells [28, 29]. Of note, all the multiplex immunotherapy panel markers in the study fall under the umbrella of IFNγ pathway. Therefore, to assess the relationship between these markers, we used Pearson correlation coefficient. As expected, *CD274 (PD-L1)* correlated with all three genes, including *PDCD1LG2 (PD-L2)*, *CD8A* and *IRF1,* which is consistent with the upregulation of IFNγ pathway reported in previous literature [10, 26, 30, 31].

The most significant limitation of this work is that our data is a single-institutional retrospective study of immunotherapy treated patients with a modest sample size. It is difficult to access clinical trial material, and hence this sort of pilot level retrospective work is required to show the potential value of new assays. Further investigation to validate the findings presented in this study are underway in collection of a validation cohort from our institution. Another limitation of this work is analyses of melanoma patients treated with either various single-agent immunotherapy or combination immunotherapy as one cohort. Future studies may address this issue by focusing on metastatic melanoma patients that received uniform treatment. Finally, in this retrospective study, we have no control or untreated arm, and thus are unable to calculate an interaction score. As such, we cannot claim predictive value for this assay and simply state that the assay is associated with outcome, without distinguishing prognostic versus predictive value.

## Conclusion

In summary, this study reports the promising association of individual immunotherapy panel markers *CD274, PDCD1LG2, CD8A*, *IRF1* and a combined *L1/L2* score (*CD274* & *PDCD1LG2*) with improved immunotherapy outcome in metastatic melanoma. The closed system mRNA approach introduced in the study has an attractive potential as an easily standardized companion diagnostic with quick turnaround time and potential for use, after further validation, as a companion diagnostic test for ICI therapy.

## Supplementary information

**Additional file 1: Figure S1.** Transcript versus protein regression. Regression of transcript versus protein for PD-L1, IRF1 and CD8 expression by nonlinear exponential growth equation.

**Additional file 2: Figure S2.** RECIST category of objective response rate and multiplex RT-qPCR immunotherapy panel markers. (A) *CD274*, *PDCD1LG2*, *CD8A*, and *IRF1* transcript expression. Data are presented as mean with standard deviation (error bars). (B) Predictive performance of *CD274*, *PDCD1LG2*, *CD8A*, and *IRF1* transcript expression by ROC curves.

## Data Availability

All data generated or analyzed during this study are included in this article and its supplementary information files.

## Abbreviations

CIs: Confidence intervals
CR: Complete response
CTLA-4: Cytotoxic T-lymphocyte antigen 4
DCR: Disease control rate
dCt: Delta cycle threshold
FDA: U.S. Food and Drug Administration
FFPE: Formalin-fixed paraffin-embedded
HR: Hazard Ratio
ICI: Immune checkpoint inhibitor
IFNγ: Interferon gamma
IHC: Immunohistochemistry
NSCLS: Non-small cell lung cancer
OS: Overall survival
PD: Progressive disease
PD-1: Programmed cell death 1
PFS: Progression-free survival
PR: Partial response
QIF: Quantitative immunofluorescence
RECIST: Response Evaluation Criteria in Solid Tumors
ROC: Receiver operating characteristic
RTq-PCR: Real-time quantitative reverse transcription polymerase chain reaction
SD: Stable disease
