# Supplementary figures and images for: Closed system RT-qPCR as a potential companion diagnostic test for immunotherapy outcome in metastatic melanoma
Source: J Immunother Cancer. 2019 Sep 18;7:254. doi: 10.1186/s40425-019-0731-9 (PMC6751819; doi:10.1186/s40425-019-0731-9)

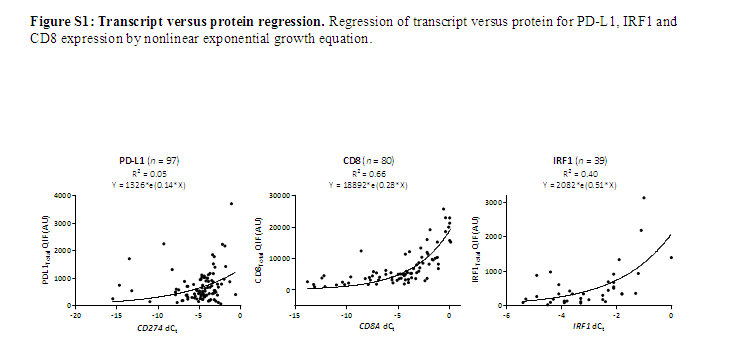

Supplement: Supplementary file 1 — Additional file 1: Figure S1. Transcript versus protein regression. Regression of transcript versus protein for PD-L1, IRF1 and CD8 expression by nonlinear exponential growth equation. [file 40425_2019_731_MOESM1_ESM.png]

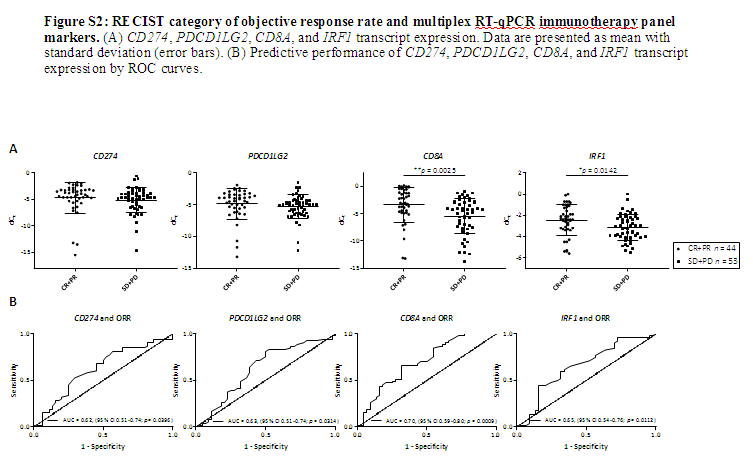

Supplement: Supplementary file 2 — Additional file 2: Figure S2. RECIST category of objective response rate and multiplex RT-qPCR immunotherapy panel markers. (A) CD274, PDCD1LG2, CD8A, and IRF1 transcript expression. Data are presented as mean with standard deviation (error bars). (B) Predictive performance of CD274, PDCD1LG2, CD8A, and IRF1 transcript expression by ROC curves. [file 40425_2019_731_MOESM2_ESM.png]
